# A Holistic Evaluation of Multivariate Statistical Process Monitoring in a Biological and Membrane Treatment System

**DOI:** 10.1021/acsestwater.3c00058

**Published:** 2023-04-06

**Authors:** Kathryn B. Newhart, Molly C. Klanderman, Amanda S. Hering, Tzahi Y. Cath

**Affiliations:** † United States Military Academy, West Point, New York 10996, United States; ‡ Baylor University, Waco, Texas 76798, United States; § Colorado School of Mines, Golden, Colorado 80401, United States

**Keywords:** wastewater treatment, fault detection, sequencing-batch reactor, membrane bioreactor, statistical learning

## Abstract

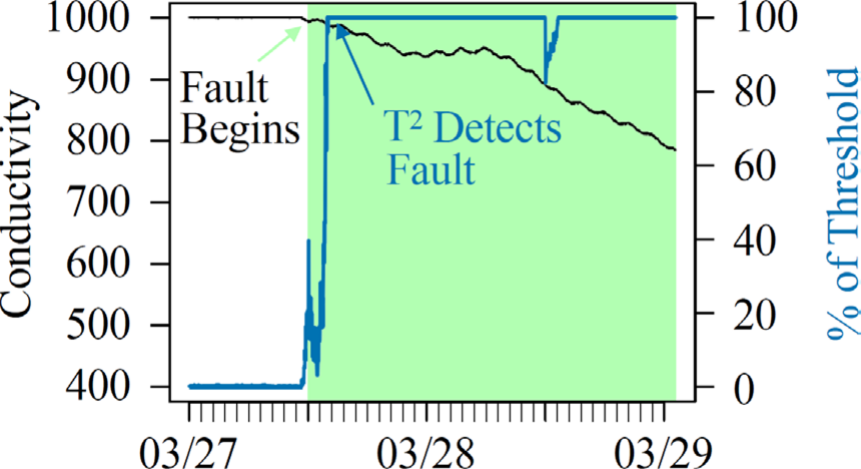

Unsupervised process monitoring for fault detection and data cleaning is underdeveloped for municipal wastewater treatment plants (WWTPs) due to the complexity and volume of data produced by sensors, equipment, and control systems. The goal of this work is to extensively test and tune an unsupervised process monitoring method that can promptly identify faults in a full-scale decentralized WWTP prior to significant system changes. Adaptive dynamic principal component analysis (AD-PCA) is a dimension reduction method modified to address autocorrelation and nonstationarity in multivariate processes and is evaluated in this work for its ability to continuously detect drift, shift, and spike faults. For spike faults, univariate drift faults, and multivariate shift faults, implementing AD-PCA on data that are subset by treatment processes and operating states with significant differences in covariates and whose model parameters use week-long training windows, moderate cumulative variance, and a high threshold for detection was found to detect faults prior to existing operational thresholds. To improve the consistency with which the AD-PCA method detects out-of-control conditions in real time, additional work is needed to remove outliers prior to model fitting and to detect multivariate drift faults in which the covariates change slowly.

## Abstract 2

A statistical process control approach is demonstrated at a decentralized wastewater treatment facility to successfully detect different types of faults without the assistance of an operator.

## 1 Introduction

Effective municipal water and wastewater treatment is imperative to protect the environment and public health.^1^ While treatment technologies continue to improve, process monitoring is often still outdated and remains heavily reliant on manual supervision and operation to detect and respond to system faults. Precision process monitoring could help water treatment plants (WTPs) and wastewater treatment plants (WWTPs) achieve their effluent quality goals (e.g., regulated nutrient and pathogen limits) in a cost-effective and reliable manner.^2^ A fault could be caused by something as innocuous as inefficient equipment performance or as severe as a process failure. However, most treatment facilities are monitored and controlled by standard supervisory control and data acquisition (SCADA) systems that use operator-determined upper and lower limits for monitoring individual process variables.^3^ Single-variable limits are established on the basis of operator experience and are inherently limited in their capacity to detect process abnormalities or faults prior to system failure. This is due to the variety of operating and environmental conditions experienced at WTPs and WWTPs throughout the year. Additionally, the multivariate nature of treatment processes weakens the single-variable monitoring paradigm because of the often changing correlation between certain process variables.^4^ In these cases, a wider range of static process limits are required to accommodate all “normal” operating conditions and avoid false alarms. While single-variable set points have a low false alarm rate (i.e., if a process variable is measured below an operator set point, then a failure to some degree involving that particular variable has most likely occurred), this approach limits process monitoring and fault detection to substantial disturbances of a single, monitored variable. To consistently meet effluent quality standards at minimum cost, more advanced process control and monitoring methods need to be integrated into WTPs and WWTPs to avoid costly process disturbances.

Statistically derived process limits are an advanced monitoring approach that has been used for early fault detection and outlier removal in industrial applications but has not yet been adapted for the water sector.^5−7^ Using previously collected data, normal operating conditions [i.e., in-control (IC) conditions] can be defined. Current data can then be compared to previously collected IC data, and abnormal [i.e., out-of-control (OC) conditions] can be identified. However, there are multiple methods of calculating the statistical thresholds associated with IC conditions, and not all methods can be directly applied to data collected at WTPs and WWTPs.

Data produced in WTPs and WWTPs frequently have missing values, contain isolated outliers, and exhibit interdependent, nonlinear, and nonstationary behavior.^8−11^ Hence, describing the treatment process using strictly mathematical models (e.g., activated sludge models or first-order decay kinetics) is often insufficient for early fault detection.^2^ It is also inappropriate to apply most standard statistical monitoring methods to WTP and WWTP data because of these features and the non-normality of the data. Changing influent quality and quantity, temperature, internal shifts in microbial ecology, and process control instability are a few causes of the observed nonstationary behavior. Furthermore, without knowledge of how the data are distributed, it is difficult to make inferences about IC or OC conditions.

Many statistical process monitoring (SPM) methods have been applied to WTP and WWTP data,^4^ including control charts^12−17^ and partial least squares.^18,19^ One important tool used in this work is principal component analysis (PCA), which is a widely used statistical method to reduce multivariate data prior to monitoring.^20^ PCA maps the underlying relationships between variables by identifying independent, linear combinations of the original variables, termed principal components (PCs), to capture as much variation in the data as possible while eliminating noise and redundancy.^21^ Data can be plotted in this lower-dimensional model space to identify clusters of similar observations or measure the distance from the observation to the model. Some applications of PCA in WTPs and WWTPs include exploratory data analysis,^22^ fault detection,^23−25^ data reconstruction,^26,27^ variable reduction for multiple regression models^28,29^ (including machine learning models^30,31^), and microbiological cluster analysis.^32^ However, the majority of the existing studies on PCA in WTPs and WWTPs has been performed at bench scale, for short periods of time,^33^ or on simulated data sets due to the complexities of the data produced from the treatment process in addition to the process itself.

To effectively apply PCA or one of many other SPM methods, the data from WWTP must be transformed such that the conditions required to appropriately apply PCA are met. For example, within a short period of time, data can be assumed to have a constant mean (i.e., stationary). Updating the PCA model with only the most recent training data (i.e., rolling window) is termed adaptive PCA and is the most popular extension of PCA for WTP and WWTP monitoring.^9,11,34,35^ However, the effectiveness of adaptive PCA is sensitive to the size of the training window. If the training window is too large, faults could be ignored because there is too much variation in the training data set.^34^ If the training window is too small, normal observations could be flagged as faults because an insufficient amount of variation is included in the training data set.^11^

To account for autocorrelation, a dynamic PCA duplicates a process variable in a data set and lags it by the number of time steps with the strongest autocorrelation for a given variable.^36^ Dynamic PCA is another common extension of PCA for monitoring industrial processes as well as WTPs and WWTPs.^26,36−39^ For most WTP and WWTP applications, a lag of a single time step is sufficient.^9^

Previous work in the literature combined the adaptive and dynamic PCA extensions to evaluate a fault event at a decentralized municipal WWTP using adaptive dynamic PCA (AD-PCA). Kazor et al. evaluated a pH fault in the biological treatment unit of a WWTP caused by a seasonal change in influent water quality and found that conventional linear AD-PCA performed much better than conventional PCA and the same as other nonlinear (and more computationally intense) adaptive dynamic dimension reduction methods (e.g., kernel PCA and local linear embedding).^9^ Additionally, Kazor et al. found that the use of nonparametric thresholds greatly reduced false alarm rates. Odom et al. further improved the AD-PCA paradigm by dividing a WWTP into multiple subsystems and incorporating “state” information for each subsystem.^40^ A “state” is defined as a set of operating parameters that produce unique conditions. At a WTP or WWTP, this is representative of how a process is being operated (i.e., process set points and limits). Statistically, this manifests as different means and covariance matrices for different operating conditions. In an activated sludge WWTP, the relationships among many monitored process variables change depending on whether the conditions are aerobic or anoxic/anaerobic [e.g., dissolved oxygen (DO) when the air blower is on or off]. In the case of Odom et al., PCA models were built for two states of blower operation using 3 days of training data. Upon application of AD-PCA to different operating states (i.e., multistate AD-PCA or MSAD-PCA), as opposed to all observations, the same pH fault evaluated by Kazor et al. was detected more quickly and consistently.

In the work presented here, single-state (SS) AD-PCA (SSAD-PCA) of Kazor et al. and multistate (MS) AD-PCA (MSAD-PCA) of Odom et al. are extended to evaluate full-scale implementation of the methods under a wide range of conditions and faults for a decentralized municipal WWTP. Kazor et al. and Odom et al. investigated only one fault using 1 week of data, but this work simulates continuous operation for large windows of time (weeks to months) that include multiple distinct fault scenarios and types (i.e., drift, shift, and spike faults over various time frames). None of these faults would have been identified with the single-variable monitoring paradigm, so they present a true challenge for the AD-PCA model to detect. Additional variations of the PCA tuning parameters were investigated to improve and explore the sensitivity of the AD-PCA model, including the division of WWTP into individual treatment units, incorporating all state information from each treatment unit, the use of training windows ranging from 1 to 14 days, and modification of the PCA model tuning parameters. Section 2 describes the WWTP studied here, AD-PCA, and the data preparation and analysis approach for each AD-PCA configuration tested. Section 3 presents the fault events to which the AD-PCA approach is applied and compares the performance across different training window sizes, metrics, and thresholds. Finally, section 4 provides lessons learned from this work and suggestions for future work to ensure the successful implementation of AD-PCA for fault detection at municipal WTPs and WWTPs.

## 2 Methods

### 2.1 Mines Park Wastewater Treatment Facility

**Figure 1:**
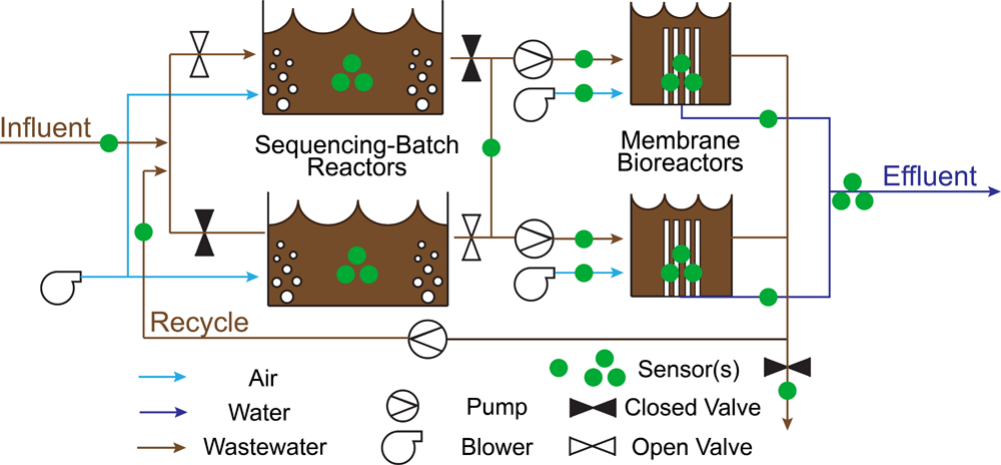
Process flow diagram of the SB-MBR integrated system, visualizing gas, liquid, and solid streams as well as the locations of real-time process measurements. A single dot indicates one process variable, and multiple dots indicate more than one variable being measured.

A sequencing-batch membrane bioreactor (SB-MBR) at the Mines Park student apartment complex (Colorado School of Mines, Golden, CO) is a coupled biological and membrane treatment system (Figure 1) treating municipal wastewater produced by the residents of 25 multifamily housing complexes for research purposes. Due to the nature of decentralized systems serving seasonal communities, the influent water quality can be highly variable and affect removal of certain contaminants. Hence, decentralized facilities such as the SB-MBR need to be closely monitored to respond to process disturbances caused by influent variability (e.g., no or impaired contaminant removal). In this work, faults that occurred in the SB-MBR are used as case studies.

The Mines Park site intercepts the municipal sewer line and diverts raw sewage to a 2500 gal (9.5 m^3^) underground holding tank where the contents are withdrawn hourly (324 gal once per hour) by a submerged grinder pump. Influent wastewater is screened (2 mm) before entering one of two partially filled 4500 gal (17 m^3^) sequencing-batch reactors (SBRs) that operate in parallel. The SBRs use an activated sludge process in alternating 2 h cycles. Influent is added to activated sludge in the SBR (10–12% exchange ratio) and is exposed to a sequence of aeration and mixing conditions to strategically transform and remove chemical and biological contaminants. Aeration is controlled by increasing or decreasing air blower output to achieve an operator-determined DO concentration set point.

In traditional SBRs, the treated water is separated from the biologically active solids by gravity. The settled solids are returned to the SBR [i.e., return-activated sludge (RAS)] and are measured as a concentration of total suspended solids (TSS). The Mines Park facility cannot fit a conventional clarifier on site and requires a higher-quality, more consistent effluent than clarifiers can achieve. Therefore, solid–liquid separation is achieved using submerged ultrafiltration (UF) membranes in the membrane bioreactors (MBRs). The PURON hollow-fiber UF membranes (Koch Membrane Systems, Inc., Wilmington, MA) used in the SB-MBR have a nominal pore size of 0.03 μm, rejecting bacteria and some viruses.^41^ Operating conditions of the SBRs and MBRs are adjusted seasonally to meet the water quality needs of users.^42−44^ The SB-MBR is controlled and monitored using a SCADA system that collects data from a variety of sensors and controls at high frequency and stores the compressed data in 1 min intervals (Table 1). Periods of time under which the conditions of the first 14 days were generally considered IC are used from February 2017 to September 2018 (Table 2). However, there were no consecutive 14 day periods in which the SB-MBR operated continuously without maintenance activities, operational changes, or changes to influent water quality. The process variables that were most variable during the 14 day training windows are included in the Supporting Information (Figures S1, S3, and S5).

**Table 1:** Monitored SB-MBR Process Variables^a^

variable	type	system	variable	type	system
BR 1 Phase	S	SBR	RAS pH	P	SBR/MBR
BR 2 Phase	S	SBR	RAS Temperature	P	SBR/MBR
BR Blower 1 Running	S	SBR	RAS TSS	P	SBR/MBR
BR Blower 2 Running	S	SBR	MBR 1 Permeate Flow	P	MBR
MBR 1 Mode	S	MBR	MBR 1 Permeate Pressure	P	MBR
MBR 2 Mode	S	MBR	MBR 2 Permeate Flow	P	MBR
MBR 1 and 2 Flux Mode	S	MBR	MBR 2 Permeate Pressure	P	MBR
MBR 1 State	S	MBR	MBR 1 Transmembrane Pressure	P	MBR
MBR 2 State	S	MBR	MBR 2 Transmembrane Pressure	P	MBR
MBR 1 Air Scour Valve Position	S	MBR	MBR 1 Air Scour Flow	P	MBR
MBR 2 Air Scour Valve Position	S	MBR	MBR 2 Air Scour Flow	P	MBR
BR 1 DO	P	SBR	MBR 1 Air Scour Pressure	P	MBR
BR 2 DO	P	SBR	MBR 2 Air Scour Pressure	P	MBR
BR Blower 1 Flow	P	SBR	MBR 1 Level	P	MBR
BR Blower 2 Flow	P	SBR	MBR 2 Level	P	MBR
Sewage Flow	P	SBR	MBR 1 Influent Flow	P	MBR
Sewage Level	P	SBR	MBR 2 Influent Flow	P	MBR
Ambient Temperature	P	SBR/MBR	MBR 1 TSS	P	MBR
BR 1 Level	P	SBR	MBR 2 TSS	P	MBR
BR 1 Temperature	P	SBR	Permeate Tank Conductivity	P	MBR
BR 2 Level	P	SBR	Permeate Tank Level	P	MBR
BR 2 Temperature	P	SBR	Permeate Tank Turbidity	P	MBR

a Variables are indicated as either state variables (type S, which indicates how a process is being operated, e.g., blower on or off) or process variables (type P, sensor measurements used to monitor or control a process). Variables included in the AD-PCA model are related to a unit process, indicated here by the system as SBR only, MBR only, or both SBR and MBR.

**Table 2:** Dates and Numbers of Observations for the Evaluated Periods of Time

time period	total no. of observations	faults
February 26, 2017, to April 3, 2017	53 226	drift (salinity), spike (salinity)
September 14, 2017, to February 28, 2018	241 915	shift (TSS)
May 1, 2018, to September 5, 2018	184 318	drift (TMP), shift (TSS)

### 2.2 Fault Events

SB-MBR fault events are evaluated and categorized as drift, shift, or spike faults by examining time-series plots and cross-referencing with operator logs. Each category of fault is discussed in detail below.

#### 2.2.1 Drift Faults

The most difficult type of fault to detect is a drift fault. Drift faults are characterized by a slow change in mean and may have an associated change in variance. The change in mean for an individual or multiple variables can be so gradual that operations staff become accustomed to the “new normal” until a variable exceeds its previously set upper and lower control limits (UCL and LCL, respectively). In this work, changes to influent quality are detected in the conductivity of the treated water. Conductivity is a surrogate measure of total dissolved solids (i.e., salts) that are not removed by conventional WWTPs so it does not have a UCL.

Operational drift faults are also common, including the accumulation of debris within or the degradation of mechanical equipment^45^ and the long-term impact of operational set point changes on water quality. The second drift fault evaluated in this work is an increased TMP as a consequence of the accumulation of solids caused by minor operational changes. A gradual increase in TMP for a membrane-based system is indicative of unsustainable operational conditions or practices that will eventually lead to membrane failure. By the time TMP exceeds its UCL, the integrity of the membranes is likely already compromised, and dramatic measures are needed to recover, such as removing membranes from the system for deep chemical cleaning or replacement.

#### 2.2.2 Shift Faults

Shift faults are distinct from drift faults in that the period over which the change occurs is much shorter than drift faults. Shift faults can occur immediately after an operational change or a system shutdown. For example, univariate shift faults are common in WWTPs when a sensor is recalibrated because the recorded value changes suddenly despite no actual change in water quality. Depending on the magnitude of the change and whether the sensor is used in a multivariate control loop, the resulting impact on operations could be either trivial or substantial. Environmental shift faults, such as a pulse injection of a contaminant in the influent wastewater, also frequently impact measurements and may be outside of the measurement range of the sensor’s prior calibration.

Two shift faults are investigated here related to changes in MBR TSS concentration. The first occurred during a prolonged shutdown in which a large volume of raw wastewater was introduced to the system, diluting the system’s TSS concentration. The second is related to sensor recalibration that resulted in a significant change in mean TSS. While both faults were consequences of operational decisions, they serve as a test of the real-world performance of AD-PCA.

#### 2.2.3 Spike Faults

Spike faults are a sudden but short-lived change in a measured value. Spike faults can occur for many different reasons (e.g., power disruption, maintenance, or cleaning) across multiple variables but can also occur in a single variable for a single instant in time or a very short period of time. For example, a power surge can cause multiple sensors to increase and decrease in value over a short period of time (seconds) simultaneously. Alternatively, a sensor may be removed from the system for service without pausing data collection. These fault events are rarely indicative of process failure, as consequential spike faults that are of concern to operators are easily detected by existing UCLs and LCLs. Rather, they are caused by intermittent sensor or operational change and should be removed from a data set prior to analysis. In the SB-MBR, permeate conductivity frequently exhibits spike fault behavior during membrane or sensor cleaning.

### 2.3 Modified PCA

The PCA model is initially constructed from a set of IC training data, and the distance metrics SPE and *T*^2^ are used to determine how well testing data maps to the PCA model. To identify the best AD-PCA configuration in this work, various tuning parameters for the method are tested. Specifically, single-state (SS) versus multistate (MS) models, variations in training window size (1–14 days), three cumulative variance percentages for PCA (80%, 90%, and 99%), and two significance levels (α = 0.01 and 0.10) are assessed.

SS includes all observations for all process variables in the SBR or MBR subsystems. MS groups observations by the operating condition of the SBR or MBR subsystems. State variables are encoded as integer values that indicate different operating conditions for treatment phases or individual pieces of equipment. These variables are used to subset the data set but are not included in the PCA model. There are 16 combinations of the SBR state variables for a given operating condition, or 16 possible states. These include the three SBR phases (fill, mix, and recirculate with MBR) and two aeration conditions (blower on or off in an individual basin). The MBR has 44 functional states due to the complex combinations of aeration and permeation. Combined, the SB-MBR has 164 state combinations. This would be an excessive number of states to monitor because of the reduction in sample size for observations occurring solely within each state. Given the effectively decoupled operation of batch (SBR) and continuous (MBR) processes, most variables are independent of the conditions in the other system. Thus, the SBR and MBR are treated as two separate systems with only a few process variables included in both data sets.

The procedures for selecting and processing the training and testing data sets (steps 1–3), applying PCA (step 4), calculating the SPE and *T*^2^ metrics (step 5), and determining if a fault has occurred (steps 6 and 7) are outlined below for SS and MS variations.

1. Identify state and process variables in the data set, and construct the input data accordingly.
  a. For SS application, include all observations of every process variable.
  b. For MS application, subset process variables to include only observations for a given state.
2. Determine the window of time for training in days (*d*_train_) and testing in days (*d*_test_), and divide the data set accordingly. In this work, *d*_train_ is varied and *d*_test_ is 1 day such that data begin and end on midnight of the day immediately following the training window.
  a. For the MS application, the number observations included for a given time period (number of observations in *d*_train_) will be inherently smaller than the SS application. The number of observations in each state’s subset of data will also likely differ unless all states have the same rate of occurrence. Here, a state is included if the number of observations for the state is at least 5 times the number of features for the subsystem. Specifically, the SBR has 14 process variables requiring 70 observations to build a PCA model, and the MBR has 24 process variables requiring 120 observations to build a PCA model. If the criterion is not met within the training window, then the observations in that state are not actively monitored for the day.
3. Scale training data to zero mean and unit variance (**X**_train_).
  a. Exclude process variables that
    i. Do not include a sufficient number of unique observations to calculate a reasonable standard deviation which may occur in the case of an offline or poorly functioning sensor.
    ii. Are offline for any significant period of the training and testing window which can be detected by a significant change in variance (e.g., non-zero to effectively zero, recording many different values to flatlined).
4. Apply PCA to training data such that **Y**_train_ = **X**_train_·**P**_train_, where **P**_train_ is the projection matrix of the ranked eigenvectors that capture the desired cumulative percent variation. In this work, 80%, 90%, and 99% cumulative variation are tested.
5. Compute the monitoring statistics, *T*^2^ and SPE, and their nonparametric thresholds, *T*^2^_α_ and SPE_α_.
  a. *T*^2^ = **Y**_train_·**Λ**_*q*_^–1^·**Y**_train_^T^, where **Λ**_*q*_ is a diagonal matrix of the eigenvalues of **P**_train_.
  b. SPE = (**X**_train_ – **Y**_train_·**P**_train_^T^)(**X**_train_ – **Y**_train_·**P**_train_^T^)^T^.
  c. Thresholds are determined by computing kernel density estimates with a Sheather–Jones bandwidth^46^ and Gaussian kernel of the training *T*^2^ and SPE values, followed by trapezoidal integration to determine the 1 – α quantiles for SPE and *T*^2^. In this work, we test α values of 0.01 and 0.10.
6. Map testing data to the PCA subspace (**Y**_test_ = **X**_test_·**P**_train_), and compute *T*^2^_test_ and SPE_test_ for **Y**_test_ and **P**_train_.
  a. *T*^2^_test_ = **Y**_test_·**Λ**_*q*_^–1^·**Y**_test_^T^, where **Λ**_*q*_ is a diagonal matrix of the eigenvalues of **P**_train_.
  b. SPE_test_ = (**X**_test_ – **Y**_test_·**P**_train_^T^)(**X**_test_ – **Y**_test_·**P**_train_^T^)^T^.
  c. For the MS application, the number of states in the training and testing data may differ. Namely, not all training states may be present in the testing data.
7. Identify IC or OC conditions.
  a. For *T*^2^, if *T*^2^_test_ < *T*^2^_α_, then the test observation is IC. If *T*^2^_test_ > *T*^2^_α_, then the test observation is OC.
  b. For SPE, if SPE_test_ < SPE_α_, then the test observation is IC. If SPE_test_ > SPE_α_, then the test observation is OC.
  c. For a simultaneous test of both SPE and *T*^2^, denoted the joint SPE–*T*^2^, if either *T*^2^_test_ > *T*^2^_α_ or SPE_test_ > SPE_α_, then the test observation is OC. Otherwise, the test observation is IC.
  d. Steps 6 and 7 are repeated for the remaining testing observations until the model retrains at midnight of each day.
8. To retrain and test, steps 1–7 are repeated with a new training data set, where *d*_train_ is held constant, the oldest *d*_test_ days are removed from the new training data set, and the IC test observations from step 7 are included in the new training data set.

For ease of interpretation, SPE_test_ and *T*^2^_test_ are transformed onto a 0% to 100% scale by dividing the test metric by their respective nonparametric thresholds and multiplying by 100. If the test metric exceeds the nonparametric threshold, it is assigned a value of 100%. Thus, when either test metric is at 100%, the observation is flagged. Due to the expected presence of noise, a single OC observation is considered “flagged”, and three consecutive OC observations are required to trigger an alarm (i.e., OC conditions for at least three consecutive minutes, which is acceptable for these relatively slow biological or physical processes).

## 3 Results and Discussion

### 3.1 Covariance State Evaluation

**Figure 2:**
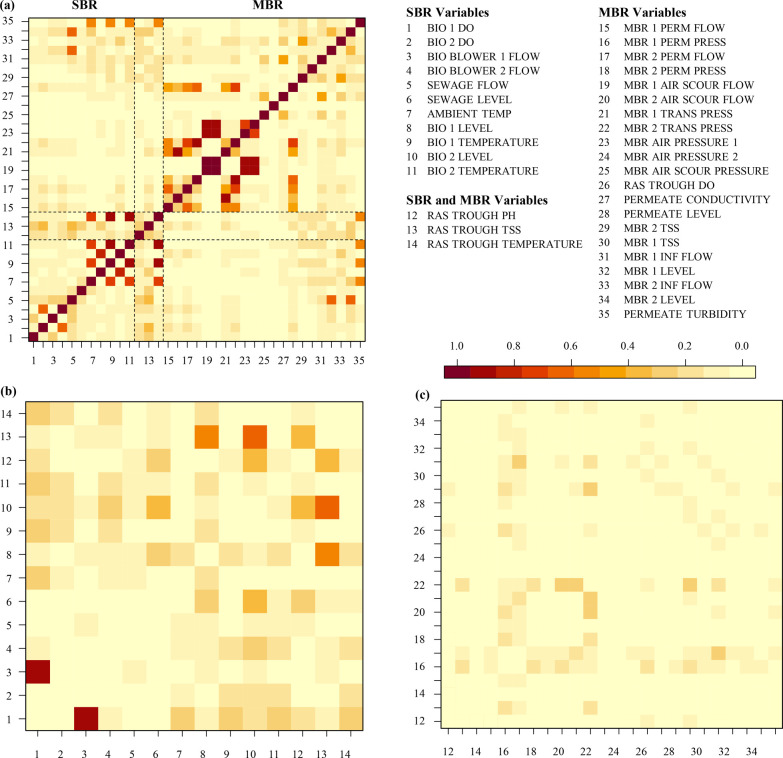
(a) Correlation matrix heat map for the SS SB-MBR system between February 26, 2017, and April 3, 2017. Zero (yellow) indicates no correlation, and 1 (red) indicates perfect linear correlation. To visualize the effect of separating training data by state, the absolute difference in correlation is plotted for the (b) two most prevalent SBR states and (c) two most prevalent MBR states.

An initial pairwise comparison of process variable covariates (Figure 2) verifies the assumption that different states have significantly different covariates with which to construct the PCA model and identifies unique relationships in the SB-MBR. For the SS SBR data set, the relationship between DO concentration and blower flow is much stronger in SBR 2 (variables 2 and 4 in Figure 2a) than SBR 1 (variables 1 and 3) in spite of the fact that the sensors, loading, diffusers, and blowers are identical for both reactors. For the SS MBR variables, permeate flow is correlated between reactors (variables 15 and 17) but not to its respective permeate pressure (variables 16 and 18), which would be expected. However, MBR permeate flow (variables 15 and 17) is related to its respective transmembrane pressure (TMP) (variables 21 and 22), from which permeate pressure is calculated (permeate pressure = atmospheric pressure – TMP). The final interesting finding from the examination of the correlations of covariates among all observations is the sensitivity to a process variable. The permeate tank level (variable 28) should remain constant except for membrane cleanings when the MBR permeate flow is reversed manually. Because only a few cleaning control indicators are recorded for the SB-MBR, it is difficult to exclude all maintenance activities that represent OC conditions. However, the unique combination of other MBR state variables (e.g., usually both air scour valves are closed manually for cleaning, which is the only instance in which both would be closed) may help remove observations that are impacted by maintenance. This illustrates the importance of separating data by state to avoid incorporating maintenance activities when defining normal operating conditions for statistical process monitoring.

If there is no difference in the correlation matrices between states, then there would be no substantial difference between the fault detection performance of SSAD-PCA and MSAD-PCA. Panels b and c of Figure 2 show the absolute difference in correlation between the two most prevalent states (i.e., those states with the most training observations) for the SBR and MBR, respectively. The most common states for the SBR are when one of the two SBRs is reacting with the blower on and the other SBR is recirculating with the MBRs with the blower off. The most common states for the MBR are when both MBRs are online and permeating but one of the two MBRs is being air scoured while the other is not. The two SBR states in Figure 2b represent 24% of the total number of training observations in the SBR system, while the two MBR states in Figure 2c represent 64% of the total training observations in the MBR system. Given the non-zero differences in correlations for many process variables in the SBR and MBR, we hypothesize that it is important to distinguish these unique states with separate PCA models, especially when attempting to detect faults in the variables whose means and variance change between states. However, the magnitude of the difference between the two MBR states is much smaller than that between the two SBR states, which would suggest that the state indicators selected for the MBR may not impact the covariates as significantly as previously thought. If there is no significant difference, then the artificial split between MBR states may weaken the nonparametric monitoring paradigm, which is sensitive to the number of observations in the training data.

### 3.2 Drift Faults

#### 3.2.1 Permeate Salinity

**Figure 3:**
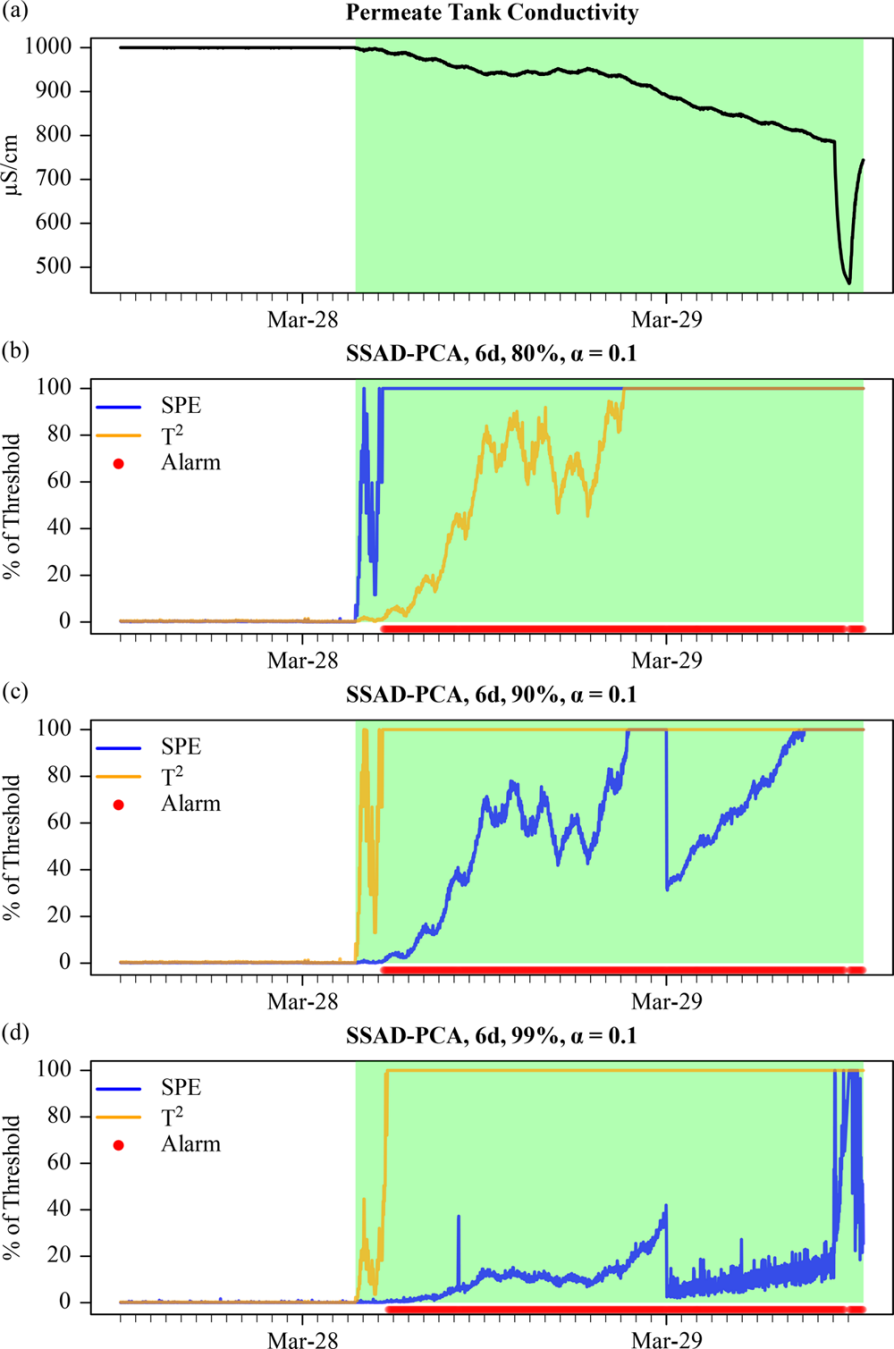
Time series and alarm plots of a drift fault where the fault period is indicated by green shading. (a) Permeate conductivity in microsiemens per centimeter. (b–d) SSAD-PCA SPE (blue) and *T*^2^ (orange) performance for 80%, 90%, and 99% cumulative variance, respectively, for the MBR subsystem during a decrease in salinity. If either of SPE or *T*^2^ causes an alarm, then a red dot is placed at the bottom of plots b–d.

A drift fault in the MBR occurred during the spring of 2017 and was caused by a change in influent quality, specifically salinity. As the UF membranes of the MBR system do not remove salts, the conductivity sensor frequently reads above the upper limit of 1000 μS/cm unless during a membrane cleaning (see Spike Faults). On March 28, the permeate conductivity began to decline at 1:45 a.m. and continued to decline until March 30 (Figure 3, top panel with suspected faulty region shaded in green), likely related to dilution during a wet-weather event. Conductivity does not have an LCL or UCL, so this fault went undetected by the existing monitoring paradigm.

The best AD-PCA configuration was SSAD-PCA with 90% cumulative variance, a 10% significance level (α = 0.10), and a 6 day training window [time to detection of 109 min (Figure 3c)]. However, multiple AD-PCA configurations achieved similar times to detection and true detection rates when training windows were ≤6 days, and no configuration had false alarms (see Table S1).

In general, SPE with a low cumulative variance performed like *T*^2^ with a high cumulative variance (80% vs 90% in panels b and c of Figure 3). This would suggest that the AD-PCA model is a reasonable but not perfect approximation of process conditions in the MBR. If AD-PCA could perfectly represent the treatment process in lower dimensional space, 99% cumulative variance would perform better than 80%. However, that is not the case for this and other faults that we examined. When all else is constant (e.g., training window size and significance level), the true detection rate for this drift fault decreases when the cumulative variance increases from 90% to 99%. This suggests that at 99% cumulative variance, the PCA model captures the variability caused by draft faults and consequently flags fewer observations. A 99% cumulative variance could be useful for fault detection schema interested in detecting only the most extreme events, especially for a robust treatment system with significant daily or seasonal variation. However, in general, a 90% cumulative variance should be considered the maximum for detecting drift faults.

The choice of cumulative variance for AD-PCA is also impacted by the total variability expected in a single state. For example, if AD-PCA can accurately represent both permeation and backwashing in a single model (in addition to other permutations of process conditions, such as air scouring for individual membrane units), then SSAD-PCA would be a better choice due to the increased number of observations used to fit the model for a set number of days in a training window. However, if a fault impacts only a few operating states, it is likely to be missed by SSAD-PCA as the observations are “diluted” by the unaffected states. For the drift fault, MS did not outperform SS. The major difference between SS and MS was the consistency of alarms (see Figure S2). This difference in MS and SSAD-PCA is to be expected when there is a frequent switching of states, as in the MBR. It is of note that the significance level did not substantially impact the alarm rate of SSAD-PCA but did delay the time to detection by approximately 20 min.

Despite the high true detection rate for these AD-PCA configurations, when retraining occurs at midnight on March 29, many MSAD-PCA configurations stop triggering alarms. Given that the PCA model is constructed using a rolling window approach, there is a loss of many older IC observations and a gain of additional variation from the next day’s observations when the model is retrained. The additional variation is included in the PCA model and increases the SPE and *T*^2^ thresholds. The amount by which the threshold increases (α decreases) is proportional to the number of IC observations lost when the rolling window moves forward and the number of OC observations included. In this situation, the OC observations are misclassified as IC prior to exceeding the threshold due to the high alarm standard. Thus, configurations of MSAD-PCA with a 1% significance level are particularly vulnerable to retraining on OC observations. However, it is important to consider the real-world scenario. If a fault consistently triggered an alarm throughout March 28, adjustments to the SB-MBR would have been made to prevent the fault from continuing into March 29, thereby making the lack of alarms into the second day less critical.

#### 3.2.2 Transmembrane Pressure

**Figure 4:**
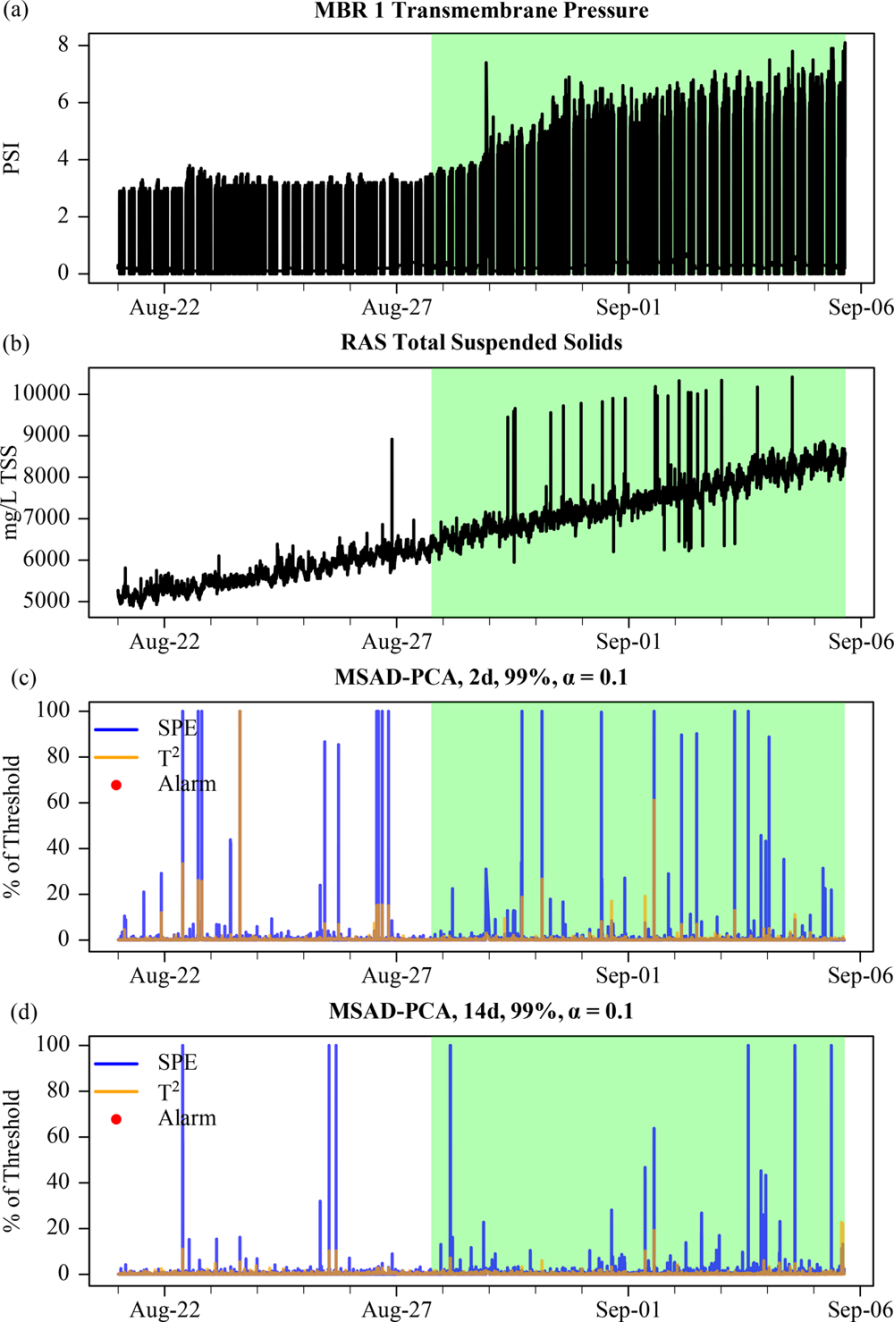
(a) MBR TMP and (b) TSS during a drift TMP fault in August 2018. MSAD-PCA metrics for (c) short (2 days) and (d) long (14 days) training periods for the MBR subsystem using 99% cumulative variance and a 10% significance level.

A TMP drift fault caused by “sludging” of the membranes occurred over the course of a month in August 2018 (Figure S4). As the permeate pump applied more vacuum to overcome the accumulation of solids on the surface of the membrane, the TMP increased. Eventually, the membranes were damaged by this high vacuum, evidenced by the increased permeate turbidity and membrane fibers present in the permeate. The change in TSS, permeate turbidity, and TMP occurred very slowly. The turbidity and TSS changed over a course of a month, while the changes to TMP were not obvious until the last week of August; the damage was not detected by operators until the first week of September (Figure 4). This fault illustrates how operator adjustments to the UCL to account for seemingly small, innocuous process changes can accumulate with catastrophic consequences. Although one could categorize this fault as a failure of human judgment, it is a normal but infrequent occurrence in the average WWTP.

The change in TSS and TMP were extremely gradual, causing small daily changes to the underlying means and covariance that went undetected by AD-PCA. The new, drifting observations were most likely mapped near the boundaries of the PCA model training subspace. Thus, it is expected and observed that the SPE metric, which measures the distance of an observation to the PCA model, would flag the most observations during the drift fault as opposed to the *T*^2^ metric, which measures the distance of an observation within the PCA model subspace (Figure 4c,d). Unlike the conductivity drift fault in the previous section, which affected a single variable over a single retraining period, the TMP drift fault affected multiple variables simultaneously over multiple retraining periods. This distinction is likely why the MS SPE flagged the most OC observations for the TMP drift fault and *T*^2^ flagged the most OC observations for the conductivity drift fault.

In addition to the distance metric, the length of the training window was the most impactful MSAD-PCA tuning parameter. As the size of the training window increased for MSAD-PCA, fewer observations were flagged [i.e., the threshold was exceeded but for fewer than three sequential observations (Figure 4d)], and SSAD-PCA did not flag any observations at all. This is likely due to the total variation present in longer training windows and with SSAD-PCA. Because the underlying trend between TSS and TMP was as expected (e.g., directly proportional), only shorter training windows would be sensitive to small changes in the covariates. The second major failing of AD-PCA in this case was the inability of SPE and *T*^2^ to consistently trigger an alarm from the OC observations in the initial testing set and the subsequent incorporation of OC observations into the training set. While the adaptation to some variation is important (e.g., seasonal changes), this presents a direct challenge to detecting multivariate drift faults such as this. In a real-world scenario, monitoring the SPE and *T*^2^ metrics by plotting (similar to Figure 4) may have been sufficient evidence for operators to investigate a change in operating conditions, even though no formal alarms were triggered. In fact, the change was so subtle that operators thought it harmless to sequentially increase the TMP UCL over the course of weeks. Had the increased TSS, TMP, and turbidity been acknowledged by operators as a fault, a more rigorous *in situ* membrane cleaning could have been initiated, and the TSS of the SB-MBR would have been reduced to avoid catastrophic membrane damage.

### 3.3 Shift Faults

#### 3.3.1 Operational Change

**Figure 5:**
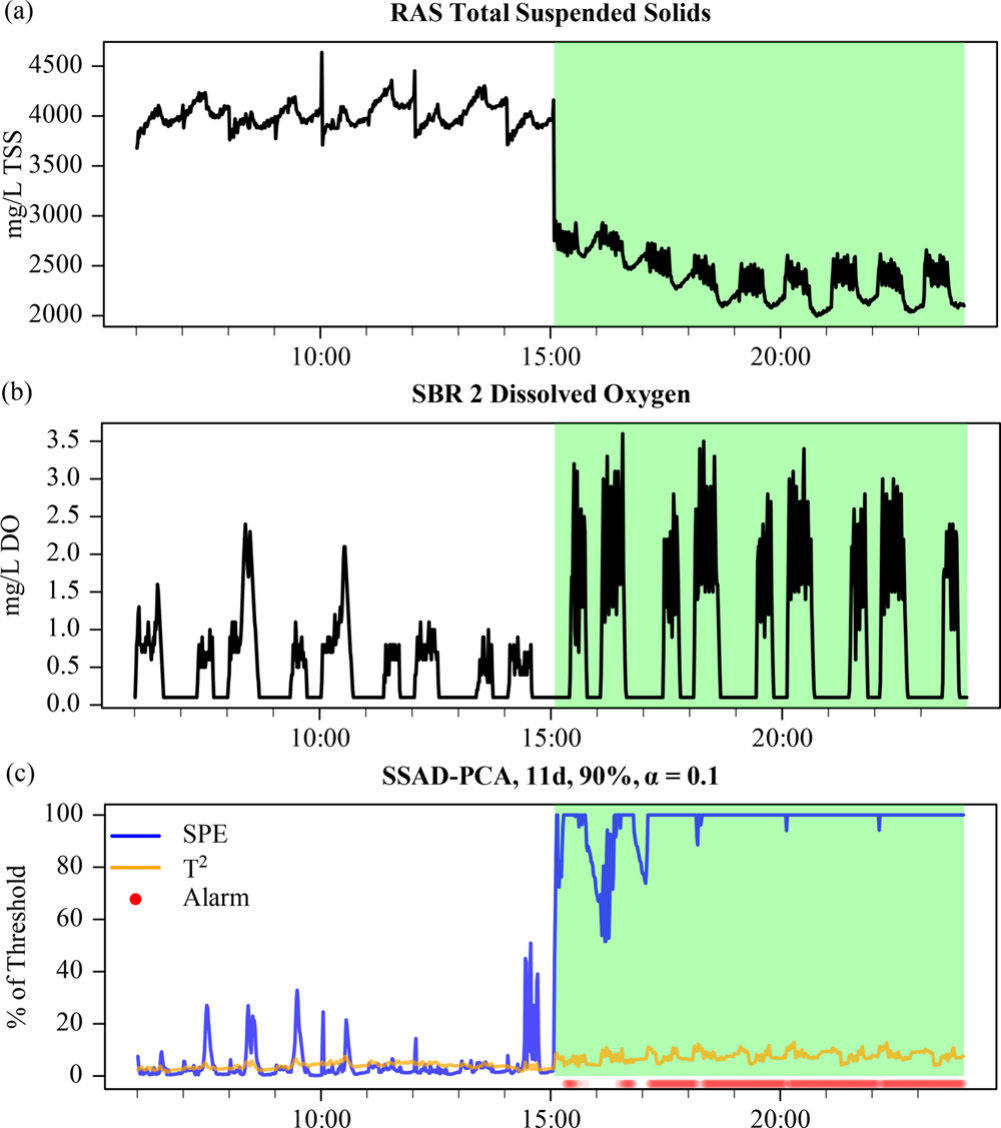
(a) RAS TSS and (b) SBR 2 DO during an overdose of raw influent. (c) SSAD-PCA for the SBR subsystem using 90% variance, a 10% threshold, and an 11 day training window. Alarms are indicated by red dots at the bottom of the AD-PCA plots.

A shift fault in which a large volume of raw wastewater was intentionally introduced into the SBRs resulted in a significantly lower TSS in the SBRs and MBRs. When the system was brought online, the TSS sensor immediately downstream of the reactors shifted downward and then very gradually increased as the more concentrated solids in the MBR system were returned to the SBR (Figure 5a).

Despite the significant change in the mean, very few of the AD-PCA configurations tested triggered alarms (Table S2). In general, SSAD-PCA with long training windows (≥8 days), moderate cumulative variance (90%), and a high level of significance (10%) were able to detect the event. MS-ADPCA triggered an alarm intermittently (Figure S6), resulting in a significantly longer time to detection and smaller true detection rate. Because the relationship between RAS TSS and DO is not fundamentally changed by the overdose event, it is possible that the majority of covariates change only slightly, similar to the multivariate drift fault discussed in the previous section. In this case, the dominance of SPE-triggered alarms would suggest that significant multivariate shift faults are not represented well in the PCA subspace. The success of SSAD-PCA with long training windows is likely an artifact of using kernel density estimation with a large number of observations, which can be required to better approximate the threshold under non-Gaussian conditions.

#### 3.3.2 Calibration

**Figure 6:**
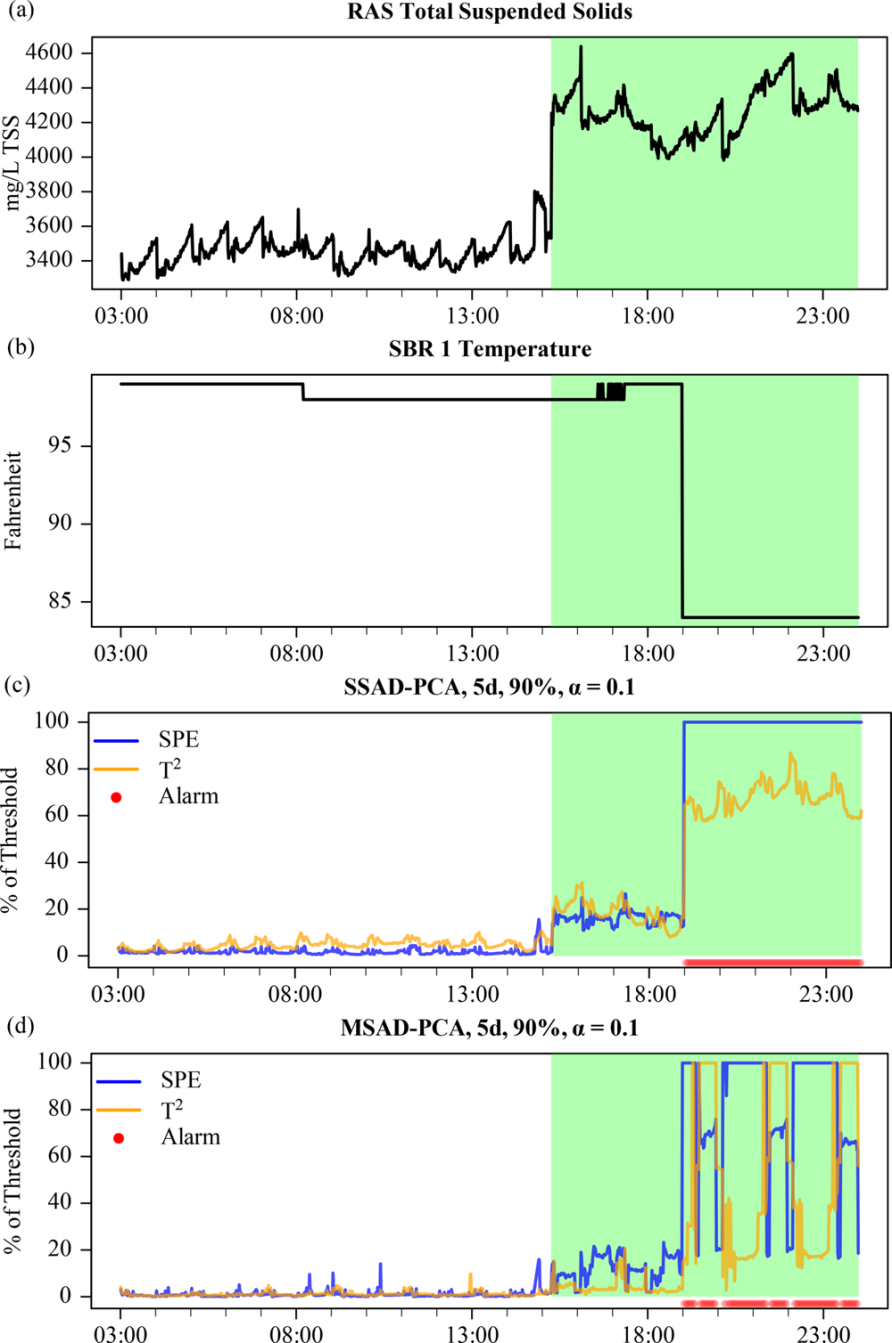
(a) RAS TSS and (b) SBR 1 temperature during a recalibration event. (c) SSAD-PCA and (d) MS-ADPCA metrics for the SBR subsystem using 90% variance, a 10% threshold, and a 5 day training window. Alarms are indicated by red dots at the bottom of the AD-PCA plots.

A second, larger TSS and temperature shift on August 14 (Figure 6a,b) caused by sensor maintenance and recalibration illustrates how AD-PCA can be used for data cleaning in addition to fault detection. In this case, SSAD-PCA generally outperformed MSAD-PCA (Table S3). The alarm response of the best MSAD-PCA configuration (Figure 6d) was less consistent than that of SSAD-PCA (Figure 6c), similar to the permeate conductivity drift fault previously discussed. Both SPE and *T*^2^ increase for the first sensor recalibration (TSS) at 1 pm but do not trigger an alarm. Once the large shift in the SBR 1 temperature occurs, then alarms are triggered across most configurations and metrics. SS consistently triggered an alarm from the calibration onward until the PCA model was retrained at midnight. For the second shift fault in temperature, MS triggered an alarm only when the air blowers in the SBRs were on, likely due to an internal temperature adjustment made by DO sensors that resulted in less air flow for the same measured DO postcalibration.

Distinct from the previously discussed faults, SPE and *T*^2^ both show significant changes as opposed to one metric dominating the other. This would suggest that multivariate shift faults are most likely to be detected by this paradigm. However, the ability of AD-PCA to detect the shift fault will depend on the variable being shifted (e.g., its impact on other variables), the magnitude of the shift, and if one or many variables shift simultaneously. In the first TSS case, isolated alarms were triggered because the impact of the shift did not alter the relationship among multiple variables. In the second case, TSS again did not trigger an alarm until an additional shift in temperature occurred. The change in temperature may have triggered the alarm as it is used to adjust DO concentration measurements.

### 3.4 Spike Faults

Spike faults are common features in wastewater treatment data sets, particularly for *in situ* instrumentation. Common causes of spike faults for *in situ* instrumentation include removal or cleaning without pausing data collection. The additional effort to pause data collection is made only when the instrument is used for control. In Figure 3, an *in situ* membrane cleaning was captured on March 29. MSAD-PCA triggered an alarm only when the cumulative variance was moderate to low (90–80%) and when the threshold was high (10%). SSAD-PCA triggered the alarm under high cumulative variance (99%), but not when the threshold was low (1%). The full set of results can be found in Table S4. For reasons previously discussed in section 3.2.1, conductivity does not impact other process variables; thus, the relatively small change in the covariates would be difficult to detect under the most stringent settings of MSAD-PCA with a high cumulative variance and a low threshold.

### 3.5 Implementation Considerations

Illustrated for the first time in this work is the matrix of choices that must be made to implement a real-time, advanced statistical process monitoring method for a complex decentralized WWTP. The majority of the literature in this area has applied a single set of tuning parameters without considering the data or system characteristics. Due to differences in how fault events affect individual process variables, it is important to consider how an ideal fault detection method would respond to an individual fault event, the fault event category, and all possible fault events. Given the range of changes to detect, it is difficult to select a single set of tuning parameters and modeling choices for every situation. From this investigation, the following recommendations should be considered for full-scale application.

1. States should be established with similar trends in process behavior (e.g., membrane permeation vs backwash) such that variables have similar correlations. First, determine monitored variables that define operational conditions (i.e., state variables). Using different permutations of these state variables, calculate the covariance matrix of the process variables and apply tools such as visualization or clustering to identify the state variables that result in significant changes in the covariance matrix. This will ensure that the maximum sample size for a given operational state is used, which influences the sensitivity of the MSAD-PCA model to process changes and outliers.
  a. For example, membrane permeation and backwash would be two important states to distinguish because of the difference in relationships between TMP and flow through the membrane. During permeation, TMP and flow are positively related. During backwash, TMP is zero and flow is negative. However, differentiating between low and high permeation rates may not be necessary for the AD-PCA model to successfully represent the process behavior because they share similar direction and magnitude in correlation for both low and high permeation rates.
2. PCA configuration values in the middle of the ranges that we tested are good initial choices for monitoring that will balance the benefits of SPE and *T*^2^. However, tuning an MS-ADPCA model to maximize flags for SPE and *T*^2^ simultaneously (as proposed in the literature) may not successfully flag all observations. Rather, one metric should be used to benchmark tuning based on the nature of the individual process and types of faults. It is suggested that cumulative variance of 90%, a significance level of 10%, and a training window of 6 days be used initially, with shorter training windows explored if events are frequently flagged but do no trigger an alarm. However, longer training windows should be used when available (i.e., outlier-free initial training window) to properly calibrate the threshold.
  a. For MSAD-PCA, the training window size needs to be long enough to include sufficient observations from less frequent, but still important, process states, generally 6–7 days. However, as the training window rolls forward to maintain a set number of days, truly OC observations that are not flagged as OC may be erroneously included in the PCA model. This can be seen in the elimination of alarms immediately after retraining and in the poor detection of multiday drift faults. If this is the case, (1) the number of states should be re-evaluated to determine if there is excessive division of training data and (2) the training window should be decreased to ≥2 days.
  b. The choice of 90% cumulative variance in the PCA captures the primary relationships between process variables and results in a more stable model response to changes. With an increase in the cumulative variance, nonlinear components may be better approximated, but it could be the same nonlinear behavior that is indicative of a fault event. A test for this condition would be if *T*^2^ exceeds a lower threshold (10% significance level) when the cumulative variance is 99% but not 80%. Alternatively, if at 99% cumulative variance the SPE is rarely small under IC conditions (20–100% of the threshold), then there is likely process variation not captured by the PCA model, and the model is overfit.
3. In addition to triggering alarms when the SPE and *T*^2^ thresholds are exceeded, operators should periodically visually inspect trends in the time series of metrics, similar to the figures presented in this work. Many of the difficult-to-detect faults (i.e., drift) are captured as increased variability in the metric as a percentage of the threshold, even if the threshold itself is not exceeded. This will also afford operators the opportunity to better understand the capabilities of AD-PCA and build trust in the tool.

## 4 Conclusion

The existing fault detection paradigm at a WWTP sets the UCL and LCL on a subset of critical process variables, which is limited and reactionary. This work explores the utility of an unsupervised statistical fault detection approach that has shown promise in the literature for biological and membrane-based treatment systems, AD-PCA. While the TMP drift fault remained challenging to detect, configurations of AD-PCA detected every other fault studied in this paper, and none of these were detected by the typical UCL and LCL thresholds. This work investigates the impact of the choice of tuning parameters (including single state vs multistate, the length of the training window, the cumulative variance of the PCA model, and the significance level of the distance metric thresholds) on the ability of AD-PCA to detect drift, shift, and spike faults and provides practical recommendations for utilities. Given that SPE measures the deviation from the PCA subspace, the low number of SPE flags for the faults evaluated in this work, and the high number of *T*^2^ flags when the thresholds are relaxed, we conclude that the combination of these two monitoring metrics with SSAD-PCA or MSAD-PCA can provide an adequate model of operating conditions in a WWTP for tracking process changes. SSAD-PCA detected most of the faults when paired with short training windows (1–6 days), a high significance level (10%), and a high cumulative variance (90%). Short fault events (minutes to hours) were more likely to be detected than long-term fault events (days to weeks) due to the intermittent flagging of OC observations by the SPE and *T*^2^ metrics.

This work is the first of its kind to assess such statistical fault detection methods under real-world conditions, applied to continuous, full-scale process data over a long period of time in which many variables required monitoring and a variety of faults occurred. We find that the inclusion of a tuned AD-PCA process monitoring program may detect multivariate environmental and operational changes currently undetected by the existing UCL and LCL fault detection method, improving the precision with which full-scale WWTPs are monitored. To further improve AD-PCA, future work should consider using methods that account for the inevitable contamination of the training window with some OC observations (e.g., robust PCA^47^), and the development of methods that are better suited to detecting long, slow drift faults, such as TMP in membrane-based systems, is also needed.

## Supporting Information Available

Figures supporting the IC training conditions and images from the SB-MBR membrane failure event (PDF)
