# Supplementary material for: A Holistic Evaluation of Multivariate Statistical Process Monitoring in a Biological and Membrane Treatment System
Source: ACS ES T Water. 2023 Apr 6;4(3):913–24. doi: 10.1021/acsestwater.3c00058 (PMC10928711; doi:10.1021/acsestwater.3c00058)
Supplement: Supplementary file 1 — ew3c00058_si_001.pdf [file ew3c00058_si_001.pdf]

SUPPORTING INFROMATION FOR:

A holistic evaluation of multivariate statistical process monitoring in a biological and membrane treatment system

Kathryn B. Newhart[1,], Molly Klanderman[2], Amanda S. Hering[2], Tzahi Y. Cath[3]*

[1] United States Military Academy, West Point, 10996, New York, USA,

[2] Baylor University, Waco, Texas, 76798, USA,

[3] Colorado School of Mines, Golden, Colorado, 80401, USA

* Corresponding author: kathryn.newhart@westpoint.edu

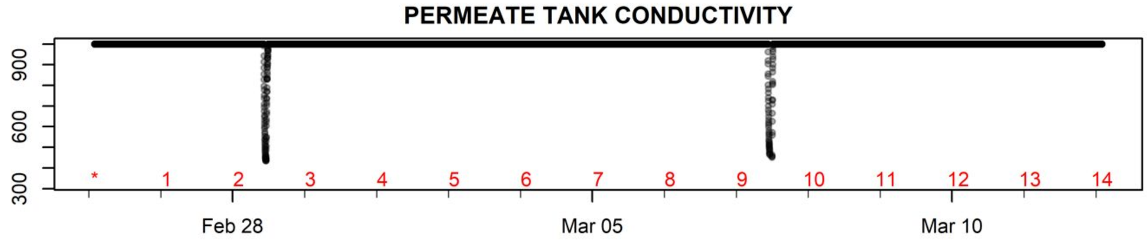

Figure S1. Training data for process variables that faulted for the 2017-02-26 dataset. The red '*' indicates the beginning of all training periods. The red number *n* indicates the end of an *n*-day training period. In this case, abnormalities are observed in the 3-day and 10-day training periods as spike faults caused by membrane cleaning operations.

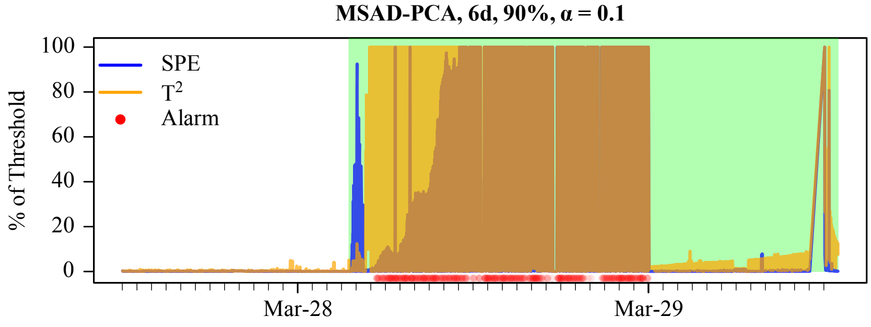

Figure S2. MSAD-PCA performance for 90% cumulative variance for the MBR subsystem during a reduction in salinity. Alarms are indicated by red dots along the bottom of the plot.

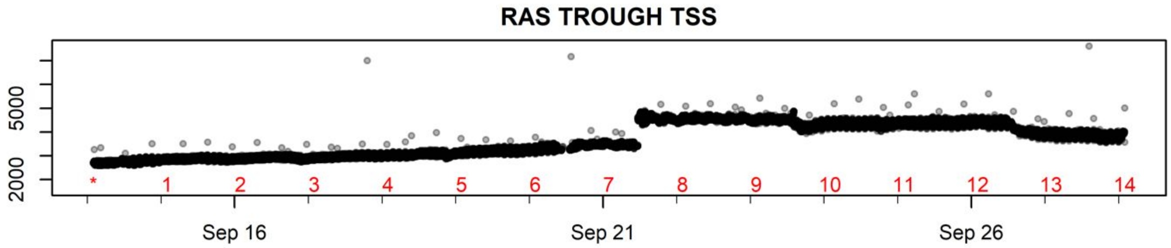

Figure S3. Training data for process variables that faulted for the 2017-09-14 dataset. The red '*' indicates the beginning of all training periods. The red number *n* indicates the end of an *n*-day training period. In this case, an abnormality is observed in the 8-day training period where a shift fault occurs due to sensor recalibration.

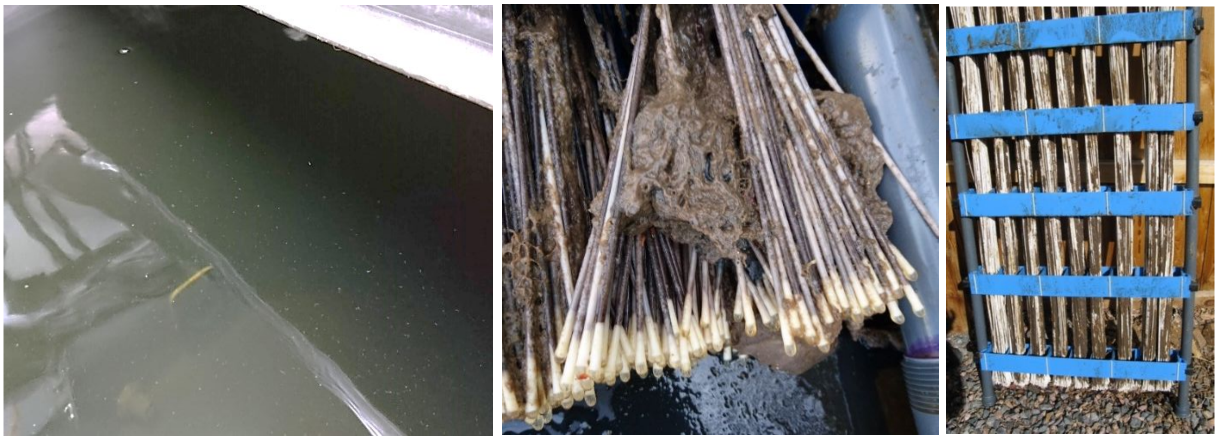

Figure S4. The membrane failure from August 2018 resulted in (a) membrane fibers visible in the permeate tank (white flecks, taken on September 5th) after (b and c) the accumulation of solids on the outside of the UF membranes caused precariously high TMP (taken on September 8th).

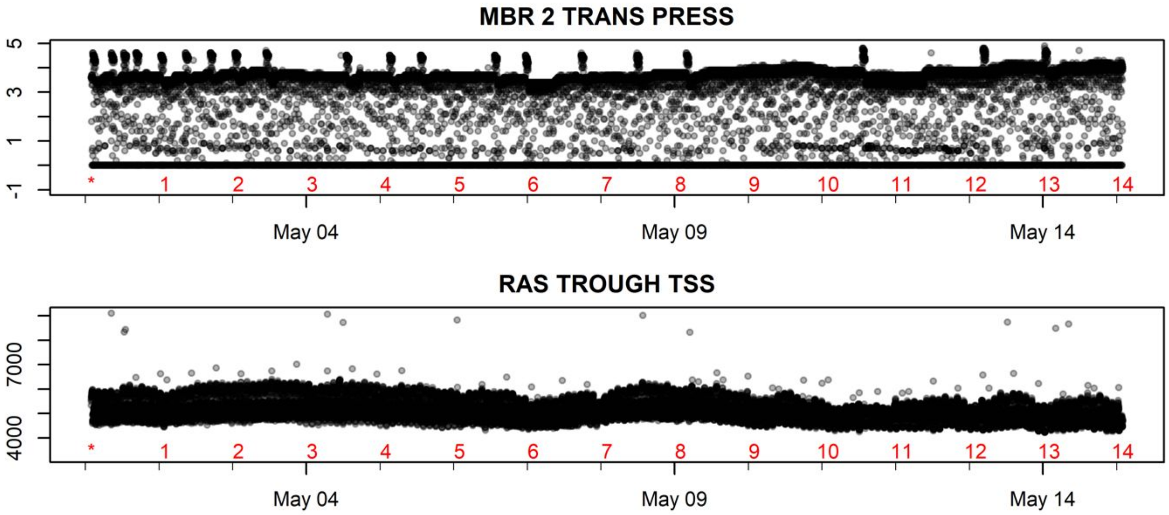

Figure S5. Training data for process variables that faulted for the 2018-05-01 dataset. The red '*' indicates the beginning of all training periods. The red number *n* indicates the end of an *n*-day training period.

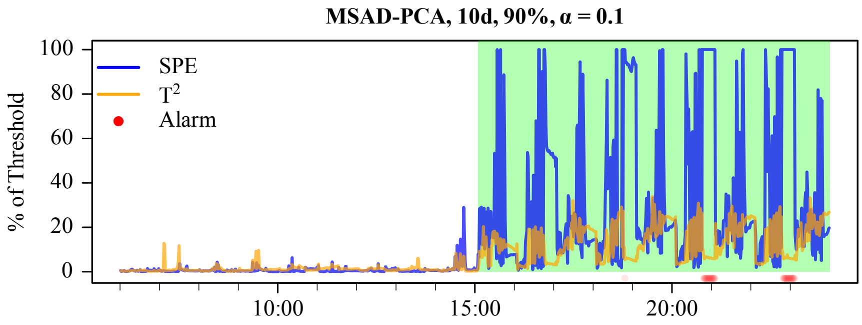

Figure S6. MSAD-PCA for the SBR subsystem using 90% variance, 10% threshold, and 10-day training window. SPE and/or T^2 alarms are indicated by red dots at the bottom.

Table S1. Results of conductivity drift fault. Training windows 7 – 14 were excluded as the fault was not detected under these conditions. False detection rate was also excluded as there were no false alarms for any AD-PCA configuration. The smallest time to detection (109 min) and the largest true detection rate (94.2%) are bolded.

Training Window (d)	Cumulative Variance (%)	Alpha	State	Time To Detection (min)	True Detection Rate (%)
1	80	0.01	SS	150	54.3
1	80	0.01	MS	337	43.8
1	80	0.1	SS	120	60.3
1	80	0.1	MS	132	89.1
1	90	0.01	SS	228	53.0
1	90	0.01	MS	365	42.1
1	90	0.1	SS	121	58.5
1	90	0.1	MS	127	89.7
1	99	0.01	SS	242	52.3
1	99	0.01	MS	383	41.1
1	99	0.1	SS	150	54.3
1	99	0.1	MS	230	50.5
2	80	0.01	MS	148	54.9
2	80	0.01	SS	164	53.8
2	80	0.1	SS	117	93.8
2	80	0.1	MS	122	91.7
2	90	0.01	MS	215	53.5
2	90	0.01	SS	224	53.2
2	90	0.1	SS	121	88.5
2	90	0.1	MS	127	85.0
2	99	0.01	MS	243	51.8
2	99	0.01	SS	255	51.6
2	99	0.1	SS	150	54.3
2	99	0.1	MS	217	53.4
3	80	0.01	MS	137	49.1
3	80	0.01	SS	138	56.0
3	80	0.1	SS	114	94.0
3	80	0.1	MS	115	51.1
3	90	0.01	MS	143	46.9
3	90	0.01	SS	214	53.8
3	90	0.1	SS	114	94.0
3	90	0.1	MS	121	50.7
3	99	0.01	SS	239	52.4
3	99	0.01	MS	254	44.0
3	99	0.1	SS	128	76.1

Training Window (d)	Cumulative Variance (%)	Alpha	State	Time To Detection (min)	True Detection Rate (%)
3	99	0.1	MS	133	48.9
4	80	0.01	SS	128	88.7
4	80	0.01	MS	144	42.3
4	80	0.1	SS	114	94.0
4	80	0.1	MS	122	45.4
4	90	0.01	SS	121	88.2
4	90	0.01	MS	133	43.9
4	90	0.1	SS	110	**94.2**
4	90	0.1	MS	117	45.8
4	99	0.01	SS	235	52.6
4	99	0.01	MS	238	40.3
4	99	0.1	SS	128	89.6
4	99	0.1	MS	133	44.0
5	80	0.01	SS	128	92.1
5	80	0.01	MS	133	40.7
5	80	0.1	SS	110	**94.2**
5	80	0.1	MS	117	42.6
5	90	0.01	SS	128	88.8
5	90	0.01	MS	133	41.8
5	90	0.1	SS	110	**94.2**
5	90	0.1	MS	113	43.0
5	99	0.01	MS	230	38.3
5	99	0.01	SS	234	52.7
5	99	0.1	SS	128	93.1
5	99	0.1	MS	133	41.9
6	80	0.01	SS	128	93.1
6	80	0.01	MS	133	24.3
6	80	0.1	SS	109	**94.2**
6	80	0.1	MS	118	25.6
6	90	0.01	SS	128	93.2
6	90	0.01	MS	133	25.1
6	90	0.1	SS	**109**	**94.2**
6	90	0.1	MS	113	25.8
6	99	0.01	MS	233	22.4
6	99	0.01	SS	234	52.7
6	99	0.1	SS	128	93.2
6	99	0.1	MS	133	25.2

Table S2. Results of TSS shift fault. AD-PCA configurations were excluded if the fault was not detected. False detection rate was also excluded as there were no false alarms for any AD-PCA configuration. The smallest time to detection (14 min) and largest true detection rate (80.4%) are bolded.

Training Window (d)	Cumulative Variance (%)	Alpha	State	Time To Detection (min)	True Detection Rate (%)
2	90	0.1	SS	151	5.4
2	99	0.1	MS	192	0.2
3	99	0.1	MS	21	0.8
8	90	0.1	SS	89	56.4
9	90	0.1	SS	142	40.4
10	90	0.1	SS	26	66.0
10	90	0.1	MS	223	7.6
11	90	0.1	SS	**14**	**80.4**
11	90	0.1	MS	343	0.4
12	90	0.1	SS	17	75.7
12	90	0.1	MS	343	0.4
13	90	0.1	SS	89	48.6
14	90	0.1	SS	89	35.7

Table S4. Results of conductivity spike fault. AD-PCA configurations were excluded if the fault was not detected. False detection rate was also excluded as there were no false alarms for any AD-PCA configuration. The smallest time to detection (0.3 min) and largest true detection rate (100%) are bolded.

Training Window (d)	Cumulative Variance (%)	Alpha	State	Time To Detection (min)	True Detection Rate (%)
1	80	0.1	SS	**0.3**	**100.0**
1	90	0.1	SS	8.3	91.3
1	99	0.1	MS	66.3	20.0
2	80	0.01	SS	21.3	35.9
2	80	0.1	SS	**0.3**	**100.0**
2	80	0.1	MS	1.3	98.0
2	90	0.1	SS	**0.3**	**100.0**
2	90	0.1	MS	1.3	98.0
2	99	0.1	SS	11.3	49.5
2	99	0.1	MS	66.3	4.0
3	80	0.01	SS	5.3	95.1
3	80	0.01	MS	68.3	10.3
3	80	0.1	SS	**0.3**	**100.0**
3	80	0.1	MS	66.3	34.5
3	90	0.01	SS	13.3	52.4
3	90	0.1	SS	**0.3**	**100.0**
3	90	0.1	MS	68.3	6.9
3	99	0.1	SS	**0.3**	**100.0**
4	80	0.01	SS	**0.3**	**100.0**
4	80	0.1	SS	**0.3**	**100.0**
4	80	0.1	MS	68.3	22.4
4	90	0.01	SS	**0.3**	**100.0**
4	90	0.1	SS	**0.3**	**100.0**
4	99	0.01	SS	30.3	22.3
4	99	0.1	SS	**0.3**	**100.0**
4	99	0.1	MS	65.3	1.7
5	80	0.01	SS	**0.3**	**100.0**
5	80	0.1	SS	**0.3**	**100.0**
5	80	0.1	MS	68.3	29.3
5	90	0.01	SS	**0.3**	**100.0**
5	90	0.1	SS	**0.3**	**100.0**
5	99	0.01	SS	26.3	31.1
5	99	0.1	SS	**0.3**	**100.0**
5	99	0.1	MS	65.3	1.7
6	80	0.01	SS	**0.3**	**100.0**

Training Window (d)	Cumulative Variance (%)	Alpha	State	Time To Detection (min)	True Detection Rate (%)
6	80	0.1	SS	**0.3**	**100.0**
6	90	0.01	SS	**0.3**	**100.0**
6	90	0.1	SS	**0.3**	**100.0**
6	99	0.01	SS	23.3	37.9
6	99	0.1	SS	**0.3**	**100.0**
6	99	0.1	MS	65.3	1.7
7	80	0.1	SS	7.3	38.8
